# Quantitative Proteomics Revealed the Molecular Regulatory Network of Lysine and the Effects of Lysine Supplementation on Sunit Skeletal Muscle Satellite Cells

**DOI:** 10.3390/ani15101425

**Published:** 2025-05-14

**Authors:** Mingxu Wang, Fan Bai, Qinan Zhao, Jianan Shi, Yutian Hao, Jindi Wu

**Affiliations:** 1 College of Food Science and Engineering, Inner Mongolia Agricultural University, Hohhot 010018, China; a1649179067@126.com (M.W.); sjn47126592@163.com (J.S.); h15771395750@126.com (Y.H.); 2 Inner Mongolia Academy of Agricultural and Animal Husbandry Sciences, Hohhot 010031, China; shmilycccp@126.com (F.B.); s.pippen.33@163.com (Q.Z.)

**Keywords:** cell differentiation, cell proliferation, lysine, quantitative proteomics of TMT, skeletal muscle satellite cells

## Abstract

Stimulating skeletal muscle satellite cells (SMSCs) with amino acids improves their proliferation and differentiation, enhancing skeletal muscle mass, thereby increasing lean meat rate. This study explored lysine (Lys)’s effects on SMSCs and their protein profiles in Sunit sheep. SMSCs were successfully isolated, assessing their survival and proliferation after Lys stimulation at varying concentrations using the CCK-8 assay. Western blotting revealed Lys-induced changes in myogenic differentiation protein expression, while immunocytochemistry detected α-Actinin and Myostatin within the SMSCs. TMT proteomics identified differentially expressed proteins, which underwent functional and interaction analyses, with RT-qPCR validating the corresponding gene expression. This study revealed that 4 mmol/L of Lys significantly boosted SMSC proliferation. A 24 h stimulation with this concentration reduced Myostatin expression, and increased MYOD1 and α-Actinin levels in the SMSCs. A proteomic analysis identified 577 differentially expressed proteins, primarily associated with lipoblast differentiation and muscle development, as highlighted by the GO enrichment analysis. A pathway analysis further demonstrated these proteins’ involvement in the autophagy–lysosome and NOD-like receptor signaling pathways. Lys enhances SMSC proliferation, differentiation, and adipogenesis in Sunit sheep, exhibiting antioxidant properties and supporting muscle stability and amino acid metabolism. It may also have anti-inflammatory, anti-pyroptotic, and proteolysis-inhibitory effects, offering insights into muscle growth mechanisms through amino acid supplementation in ruminants.

## Simple Summary

Research has demonstrated that stimulating skeletal muscle satellite cells with amino acids enhances their growth and differentiation, ultimately boosting lean meat production. This study employed methods such as the CCK-8 assay, Western blot, and Tandem Mass Tags proteomics to establish that 4 mmol/L of lysine significantly improved skeletal muscle satellite cell proliferation, reduced Myostatin expression, and increased MYOD1 and α-Actinin levels. Additionally, lysine displayed antioxidant properties, supported muscle stability, facilitated amino acid metabolism, and promoted adipogenesis. These findings provide valuable insights into the muscle growth mechanisms driven by amino acid supplementation in ruminants.

## 1. Introduction

Small ruminants, especially native breeds, play a pivotal role in the socio-economic livelihoods of a considerable portion of the human population in tropical regions. These animals serve as essential providers of meat, milk, wool, and hides, thereby contributing significantly to food security and rural incomes. Improving the economic and biological efficiency of small ruminant production hinges on increasing both productivity and reproductive performance [1]. Enhanced reproductive outcomes can be achieved through better nutrition, targeted breeding strategies, and advanced reproductive technologies such as artificial insemination and embryo transfer. Integrating these practices enables small ruminant breeders to boost flock productivity, reinforce food security, and drive the economic prosperity of rural communities [2].

Sunit sheep, as one of the excellent breeds in Inner Mongolia, are a coarse sheep bred for meat use. They have the characteristics of a large size, tender meat, moderate fat content in their meat, containing a variety of minerals beneficial to the human body, and rapid development and growth [3]. Meat quality is one of the key factors in lamb production, and the factors that affect meat quality are often regulated by many genetic or nutritional controls. Lys, as the first limiting essential amino acid of grain-based mammals, promotes the growth of mammalian skeletal muscle by increasing protein synthesis [4,5]. However, protein synthesis is regulated by genes in the nucleus, and the greater the number of nuclei in a muscle fiber, the higher the efficiency of protein synthesis [6], so that the conversion of proteins is extended to the conversion of cells. In this conversion process, the SMSCs located under the muscle fiber plate proliferate, differentiate, and fuse muscle fibers to form a new muscle nucleus, thereby causing the enlargement of skeletal muscle fibers and the mutual transformation of skeletal muscle fiber types. Some studies have found that Lys acts as a signaling molecule to activate SMSCs to promote the growth of skeletal muscle [7,8] and acts as a material for protein synthesis during the growth process [9,10]. However, the functional development of SC is complex and there are still many uncertainties to be investigated [11]. In recent years, proteomics has emerged as an important and powerful post-genomic tool in the field of meat science [7], which represents the analytical methods that allow for the analysis of the proteins (proteomes) of cells or tissues under certain given conditions. And proteomics is able to address the interactions of different proteins in order to better understand the results of these interactions [12]. Previous studies, using an iTRAQ analysis, have found that Lys plays another role as a signaling molecule that activates SMSCs to promote skeletal muscle growth in piglets [13]. In this study, the publicly available Sunit sheep proteomic database created a new way to study the regulatory mechanism of Lys on SMSCs and how it affects mutton quality [14].

We designed an experiment to screen the optimal concentration of Lys through cck8 and induce Sunit sheep SMSCs with the optimal concentration of Lys to observe the changes in cell proliferation and cell cycle. Furthermore, this study utilized TMT quantitative proteomics technology to identify the differential proteins in SMSCs and the pathways in which they are enriched. For the first time, the molecular regulatory mechanism underlying the addition of Lys to SMSCs in Sunit sheep has been thoroughly analyzed.

## 2. Materials and Methods

### 2.1. Animals and Cells

All samples (*n* = 3) were obtained from Sunit fetal sheep that were incidentally found pregnant at the slaughterhouse. The fetal sheep were collected and soaked in 75% alcohol for 3–5 min. After soaking, the fetal sheep were dissected, and their longissimus dorsi muscle tissue was extracted. The tissue was soaked in PBS with 1% penicillin (TransGen Biotech, Beijing, China) and left for 30 min at 4 °C. The tissues were placed on a glass plate in a safety cabinet, cut into small pieces, and transferred into a centrifuge tube with collagen II (Saiguo, Guangzhou, China) and a DMEM medium (Hyclone, South Logan, UT, USA). The mixture was slowly digested in a 37 °C shaking incubator (ZHICHENG, Shanghai, China) for 45 min. After digestion, the mixture was centrifuged at 2000× *g* for 3 min at 4 °C, and the supernatant was discarded.

Trypsin (Gibco, Grand Island, NY, USA) was added to the precipitate, mixed thoroughly, and digested again for 30 min at 37 °C. Impurities were filtered with a 100-mesh cell screen. The mixture was centrifuged at 2000× *g* for 3 min at 4 °C, and the supernatant was discarded. The precipitate, containing cells, was resuspended in an appropriate amount of a complete medium, and the cells were carefully mixed. The cells were then divided into several culture bottles and cultured at 37 °C with 5% CO_2_ (Heal Force, Shanghai, China).

The medium was changed approximately every 12 to 36 h, depending on medium consumption and cell growth. When the cells reached 70–80% density, they were ready for passage. The medium was discarded, and the cells were washed 2–3 times with 3 mL of PBS containing 1% antibiotic. The cells were digested with trypsin at 37 °C for 3 min. When observed under an inverted microscope, the SMSCs began to detach, indicating the end of digestion. Digestion was stopped by adding twice the volume of a 10% horse serum (Solarbio, Beijing, China) medium. The cells were then centrifuged at 1500× *g* for 5 min at 4 °C, the supernatant was discarded, and the cells were resuspended in a complete medium. Finally, the cells were divided into several culture bottles and cultured at 37 °C with 5% CO_2_.

Fifth-generation Sunit sheep SMSCs were fixed with 4% paraformaldehyde (Biosharp, Anhui, China) for 30 min, permeabilized with 0.1% Triton X-100 (Saiguo, Guangzhou, China) at room temperature for 1 h, and blocked with 5% BSA (Solarbio, Beijing, China). The cells were then incubated overnight with primary antibodies, a Paired box protein (PAX7) (1:200, Abcam, Cambridge, MA, USA), and a Myo-differentiating protein (MYOD1) (1:200, Biorbyt, Cambridge, UK). After washing off the primary antibodies, the cells were incubated with an HRP-conjugated secondary antibody (1:1000, Affinity Biosciences, Cincinnati, OH, USA) at 37 °C for 2 h. The cells were subsequently stained with a DAPI (Gibco, Grand Island, NY, USA) solution for 5 min and observed under a confocal microscope (Nikon ECLIPSE Ti-U, 770, Midori, Otawara, Japan).

### 2.2. Lys Concentration Screening

SMSCs were treated with a Lys (Solarbio, Beijing, China) medium at eight concentration gradients (0 μmol/L, 1 mmol/L, 2 mmol/L, 4 mmol/L, 8 mmol/L, 16 mmol/L, 32 mmol/L, 64 mmol/L) for 24 h, along with a blank group and a control group, the effect of Lys on the survival rate of these cells was determined using the CCK-8 method. OD values were measured at a wavelength of 450 nm to determine the optimal Lys concentration.

### 2.3. Cell Proliferation Experiment

Fourth-generation SMSCs were seeded into a 96-well cell culture plate and allowed to adhere and grow for 24 h. They were then divided into two groups: the CON group, cultured in a complete medium without Lys (0 mmol/L), and the LYS group, cultured in a complete medium with 4 mmol/L of Lys. The OD_450_ values were measured at 24, 48, 72, 96, and 120 h after Lys treatment in the SMSCs using the CCK-8 method.

### 2.4. Western Blot

The SMSCs were transferred to a centrifuge tube and centrifuged at 1500× *g* for 10 min. The supernatant was discarded, and a PBS buffer was added before centrifuging again at 1500× *g* for 5 min. A Lysis Buffer (containing 1 mL Lysis Buffer, 5 μL phosphatase inhibitor, 1 μL protease inhibitor, and 10 μL PMSF) (Solarbio, Beijing, China) was added to the cells. The mixture was transferred to a new centrifuge tube and shaken at 4 °C to lyse the cells. The solution was then centrifuged at 20,000× *g* for 15 min at 4 °C. The supernatant, containing the whole protein extract, was collected and stored at −80 °C.

Protein concentration was determined using the BCA method (Beyotime, Shanghai, China). Proteins were separated by electrophoresis on 8–12% SDS-PAGE gels (Solarbio, Beijing, China) at 80 V for 200 min and transferred to a polyvinylidene fluoride (PVDF) membrane (Merck Millipore, Billerica, MA, USA) at 240 mA for 90 min. The membrane was blocked with 5% skim milk (BD, San Jose, CA, USA) at 4 °C overnight.

After blocking, the membrane was washed three times with 1× TBST (Tris-HCl, NaCl, Tween) for 5 min each time. The membrane was then incubated with the primary antibody at 4 °C overnight. Following primary antibody incubation, the membrane was washed four times with 1× TBST for 15 min each. The membrane was then incubated with the secondary antibody (Goat Anti-Rabbit IgG HRP) (Affinity Biosciences, Cincinnati, OH, USA) at room temperature for 2 h. After secondary antibody incubation, the membrane was washed five times with 1× TBST for 15 min each. The membrane was rinsed with a developer (Thermo, Waltham, MA, USA) and exposed to visualize the protein bands.

The resulting bands were analyzed using ImageJ (version 1.53o) analysis software, and histograms were generated using GraphPad Prism 8.0.

### 2.5. Immunocytochemistry

Fourth-generation SMSCs were seeded into confocal dishes and allowed to adhere and grow for 24 h. The cells were then divided into two groups: the CON group (cultured in a complete medium) and the LYS group (cultured in a complete medium supplemented with 4 mmol/L of Lys) for an additional 24 h.

After discarding the culture medium, the cells were fixed with 4% paraformaldehyde for 20 min, permeabilized with 0.5% Triton X-100 at room temperature for 20 min, and blocked with 5% BSA at room temperature for 30 min. Next, the primary antibodies Mouse Anti-α-Actinin (Sarcomeric) and Anti-GDF8/Myostatin were diluted at a 1:200 ratio and added dropwise, followed by overnight incubation at 4 °C.

The primary antibodies were washed three times with PBS, then HRP-conjugated secondary antibodies (diluted 1:100) were added dropwise and incubated at room temperature in the dark for 1 h. The secondary antibodies were washed three times with PBS, followed by incubation with DAPI for 15 min in the dark. After washing five times with PBS, the cells were mounted with glycerol and observed under a confocal microscope.

### 2.6. TMT Quantitative Proteomics

An appropriate amount of lysate (8 M Urea, 1% protease inhibitor mixture) was added to extract the total protein from the samples. Protein concentration was determined using a BCA kit. A total of 100 micrograms of protein from each sample were taken, and disulfide bonds were reduced by adding DTT, reacting at 30 °C for 60 min. IAM was added to block free sulfhydryl groups, reacting at room temperature for 45 min in the dark. The proteins were precipitated with acetone and rinsed. TEAB was added, and the proteins were dissolved by an on-ice ultrasound.

The proteins were enzymatically digested with trypsin at 37 °C overnight. The peptides were desalted using a C18 SPE column, and the eluted peptides were dried using a vacuum concentrator. Additionally, 100 µg peptide segments from each sample were labeled according to TMT reagent instructions, redissolved in a liquid A solution, mixed, and centrifuged at 20,000× *g* for 2 min. The supernatants were loaded onto C18 columns, and reverse gradient separation was performed by high-performance liquid chromatography under alkaline conditions. The eluted peptide components were combined and dried using a vacuum concentrator. The pre-separated components were dissolved in liquid A, centrifuged at 20,000× *g* for 2 min, and the supernatants were transferred to sample vials. The LMS system analyzed each component for 1 h, with approximately 2 µg of loading.

Mass spectrometry raw files were retrieved using MaxQuant (v.1.5.2.8). Search parameters included species-specific protein databases, Trypsin/P digestion, TMT quantification, a maximum of 2 missed cleavages, an FDR ≤ 0.01, a primary ion mass error tolerance of 10 ppm, and a secondary fragment ion tolerance of 0.02 Da. The fixed modifications included cysteine alkylation; the variable modifications included methionine oxidation, deamidation (NQ), and N-terminal acetylation.

Gene Ontology (GO) annotations were sourced from the UniProt-GOA database “http://www.uniprot.org/ (accessed on 20 December 2023)”. Protein IDs were converted to UniProt IDs, matched to GO IDs, and the corresponding information was retrieved. If the information was not found in UniProt-GOA, InterProScan was used to predict GO functions. Proteins were classified based on cellular composition, molecular function, and physiological processes.

### 2.7. Real-Time Fluorescent Quantitative PCR Validation Assay for Differentially Expressed Proteins

RNA was extracted from the SMSCs using TRNzol Universal (Tiangen, Beijing, China), and the total RNA was reverse-transcribed into cDNA using the HiScript^®^ II Q RT SuperMix for the qPCR (+gDNA wiper) kit (Vazyme, Nanjing, China). Real-time fluorescent quantitative PCR amplification was performed according to the ChamQ Universal SYBR qPCR Master Mix kit (Vazyme, Nanjing, China) instructions, with a total reaction volume of 20 μL.

The amplification program included the following: 95 °C for 30 s, 95 °C for 10 s, and 60 °C for 20 s, repeated for 38 to 40 cycles. The dissociation curves were analyzed using a real-time fluorescent quantitative PCR instrument to obtain the Ct values for each gene. The relative expression levels of each gene were calculated using the 2^−ΔΔCt^ method. β-actin was used as the internal reference gene in this experiment. The primers for the target and internal reference genes were designed based on sequences provided by NCBI (Table 1) and synthesized by Sangon Biotech (Shanghai) Co., Ltd. (Sangon Biotech, Shanghai, China).

### 2.8. Statistical Analysis and Processing of the Data

Data are expressed as mean ± standard error (SEM). The results of the control group, Lys-deficient group, and LYS group (*n* = 3) were statistically analyzed using one-way analysis of variance (ANOVA) and the Tukey test. Data were analyzed using GraphPad Prism 8.0 software. A difference of *p* < 0.05 was considered statistically significant, while *p* < 0.01 was considered extremely significant.

## 3. Results

### 3.1. Effect of Lys Treatment on Survival Rate of SMSCs

**Figure 1:**
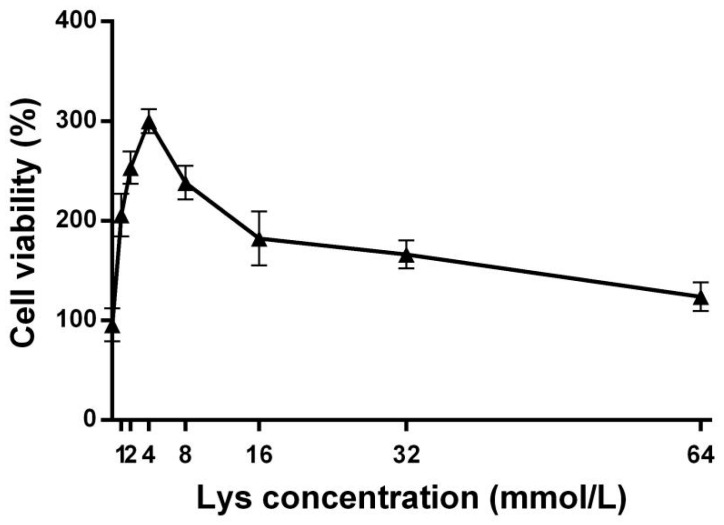
Effects of different concentrations of Lys on the survival rate of SMSCs.

After treating SMSCs with different concentrations of Lys (0, 1, 2, 4, 8, 16, 32, 64 mmol/L) [15], the effect of Lys on the survival rate of these cells was determined using the CCK-8 method. As shown in Figure 1, Lys significantly improved cell survival rate compared to the control group (*p* > 0.05). When the concentration of Lys was less than 4 mmol/L, the cell survival rate gradually increased with the increase in Lys concentration. When the Lys concentration exceeded 4 mmol/L, the cell survival rate began to decline, but it remained higher than that of the control group. Therefore, 4 mmol/L of Lys was selected as the treatment concentration for subsequent experiments. 

### 3.2. Effect of Lys on the Proliferation of SMSCs

**Figure 2:**
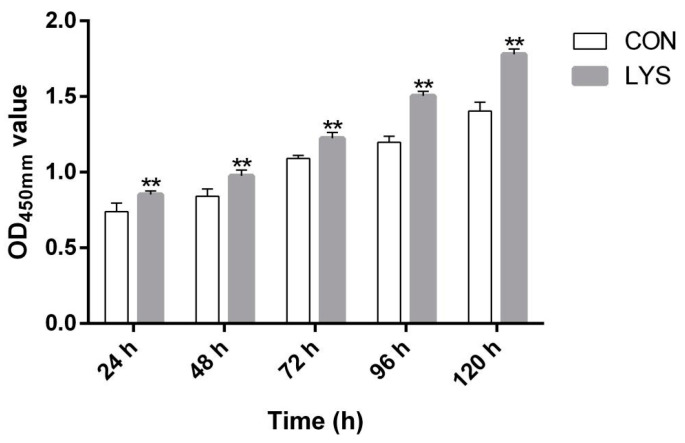
Effects of Lys on skeletal muscle satellite cell proliferation. “**” was considered *p* < 0.01.

The CCK-8 results, as shown in Figure 2, indicate that with the increase in the duration of Lys induction in SMSCs, the OD_450_ values in the LYS group gradually increased compared to the CON group. Furthermore, the OD_450_ values in the LYS group were significantly higher than those in the CON group (*p* < 0.01), indicating that the SMSCs significantly proliferated under the stimulation of Lys (*p* < 0.01). 

### 3.3. The Effect of Lys on Myogenic Differentiation Protein Expression

**Figure 3:**
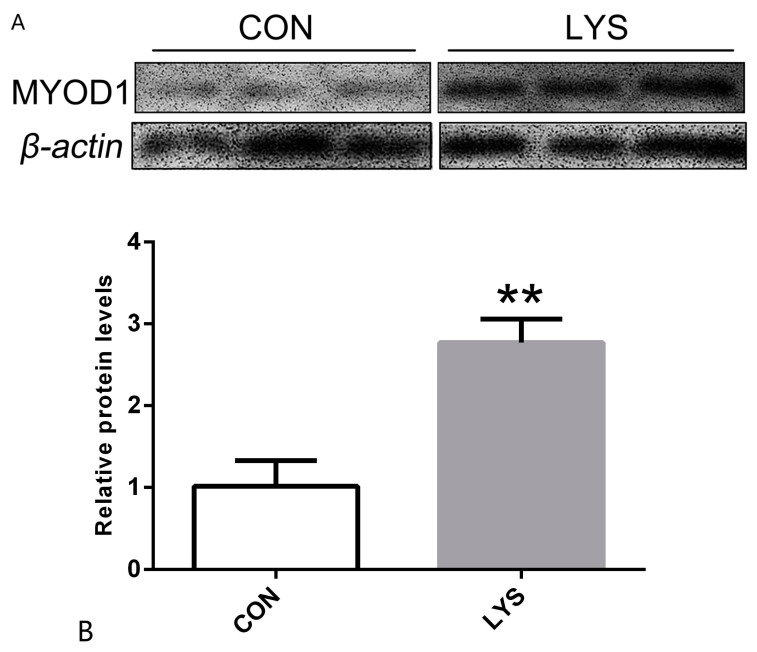
(**A**) The 24 h MYOD1 protein bands were differentiated. (**B**) The relative expression of MYOD1 protein at 24 h of differentiation. “**” was considered *p* < 0.01.

The results, illustrated in Figure 3, indicate that after 24 h of Lys induction in SMSCs, the expression of the myogenic differentiation protein MYOD1 was significantly upregulated in the Lys group compared to the control group (CON) (*p* < 0.01). Since MYOD1 is crucial for skeletal muscle development, these findings suggest that Lys not only promotes the proliferation and differentiation of SMSCs but also contributes to an increase in skeletal muscle mass.

### 3.4. The Effect of Lys on the Expression of α-Actinin and Myostatin in SMSCs

**Figure 4:**
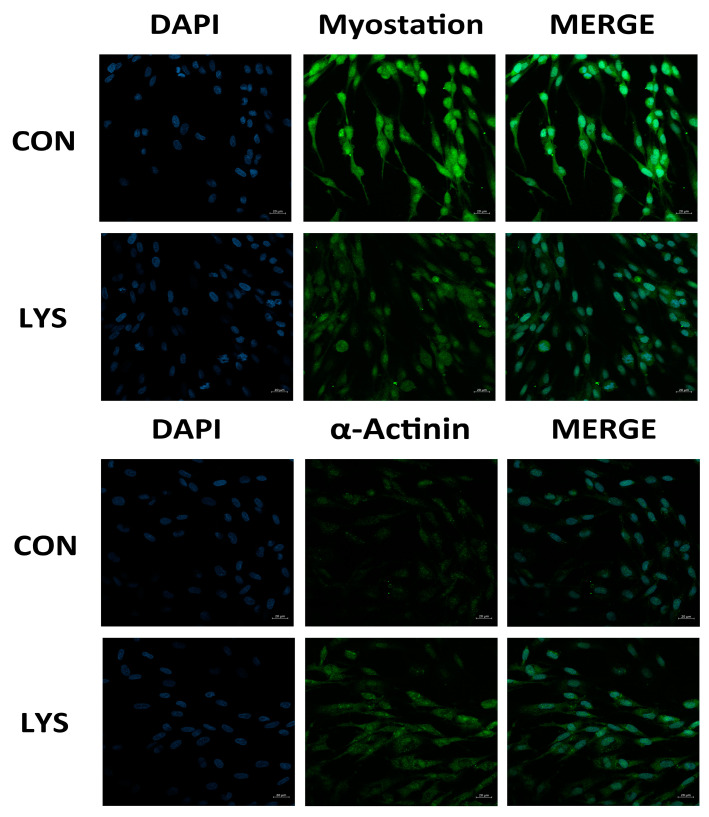

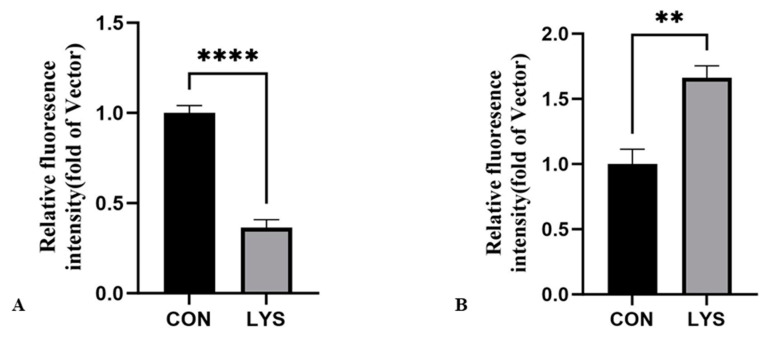
Immunofluorescence identification results of the SMSCs in the CON group and LYS group (20× magnification). (**A**) Average fluorescence intensity of myostatin expression. (**B**) Average fluorescence intensity of α-actinin expression. “**” was considered *p* < 0.01; “****” was considered *p* < 0.0001.

Immunocytochemical techniques were used to detect the expression of α-Actinin and Myostatin in the SMSCs of the control (CON) and LYS groups. Immunofluorescence images revealed that both the Myostatin and α-Actinin proteins exhibited immunopositive expressions. Green fluorescence was observed in the cytoplasm, while blue fluorescence in the nuclei was revealed by DAPI counterstaining (Figure 4).

In comparison to the CON group, Myostatin expression was profoundly reduced in the LYS group (*p* < 0.0001). Conversely, the expression of α-Actinin was significantly enhanced in the Lys-induced SMSCs, showing a difference from the control group (*p* < 0.01).

### 3.5. TMT Quantitative Proteomics Analysis

#### 3.5.1. TMT Quantitative Proteomics Identification

**Figure 5:**
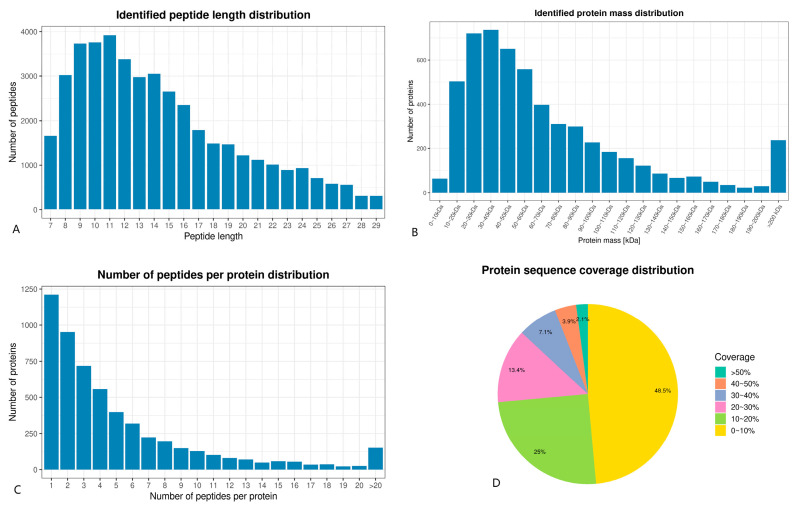
(**A**) Identified peptide length distribution. (**B**) Identified protein mass distribution. (**C**) Number of peptides per protein distribution. (**D**) Protein sequence coverage distribution.

Most of the peptides were 8–16 amino acids in length, with the largest percentage of peptides being 9–12 amino acids long (Figure 5A). This distribution aligns with the typical pattern based on trypsin digestion and HCD fragmentation mode, meeting the quality control requirements for mass spectrometry identification [3]. The TMT technique identified 64 low molecular weight proteins (Mw < 10) and 1088 high molecular weight proteins (Mw > 100) (Figure 5B).

The peptide number distribution indicated that 77.5% of the identified proteins contained at least two peptides. Most proteins consisted of fewer than 10 peptides, with the number of proteins decreasing as the number of matching peptides increased (Figure 5C). This precise distribution supports its plausibility. The protein sequence coverage results showed that over 51.5% of the identified proteins had sequence coverage higher than 10% (Figure 5D).

#### 3.5.2. Statistical Analysis of Differential Proteins

In this study, 5607 proteins were identified in SMSCs of the longest dorsal muscle, with 5172 being quantifiable. For quantitative analysis, the relative quantitative values of each sample were calculated based on the signal intensity of the labeling reagents in the mass spectra. The relative protein expressions in the experimental group were compared with the control group according to the experimental design scheme.

The quantification of multiple replicate experiments was combined by averaging the quantification ratios between the experimental and control groups. Differentially expressed proteins were screened based on relative quantification values greater than 1.2 or less than 1/1.2 and a statistical *t*-test *p*-value of less than 0.05. The proteins with a relative quantification value > 1.2 were considered significantly upregulated, while those with a relative quantification value < 1/1.2 were considered significantly downregulated.

**Figure 6:**
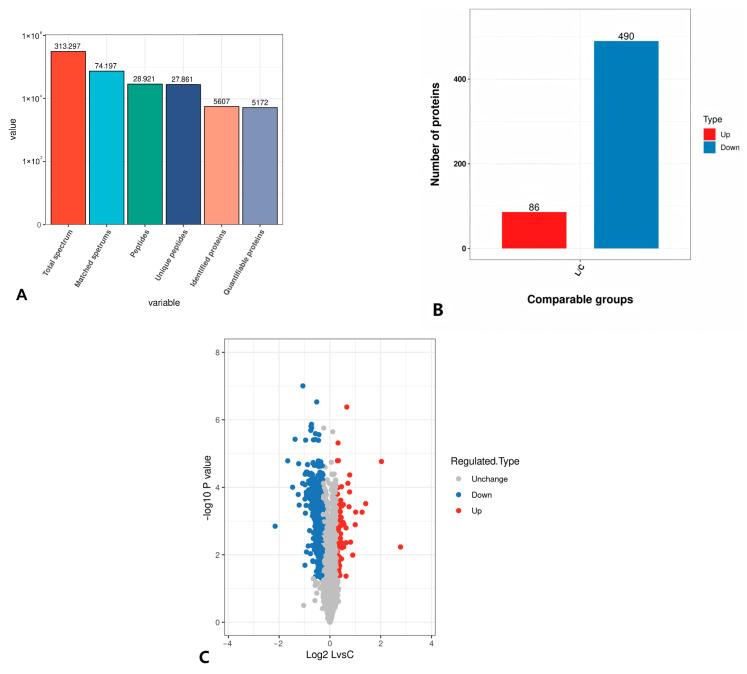

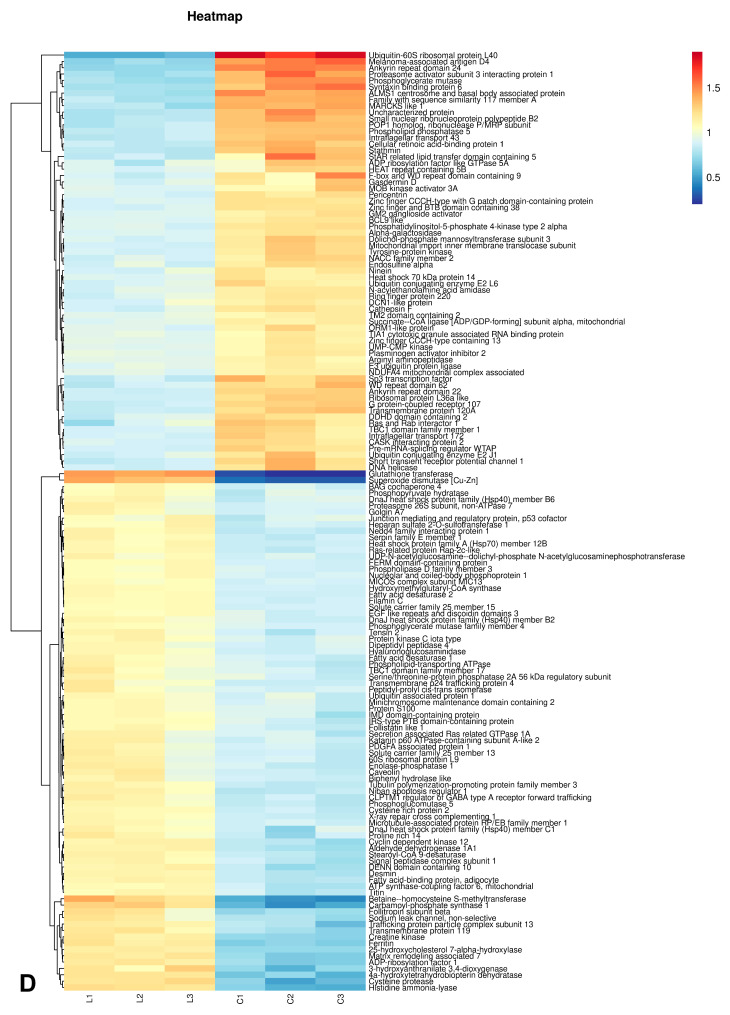
Statistical analysis results of differential proteins. (**A**) Protein expression identification and quantitative results. (**B**) Statistics of differential proteins. (**C**) Volcano map of differential proteins. (**D**) Clustering heat map.

The final screening identified 576 differentially expressed proteins, including 86 upregulated and 490 downregulated proteins (Figure 6A). A volcano plot was created using the relative quantitative values of the proteins after a Log2 transformation on the horizontal axis and the *p*-value of the significance test after a –Log10 transformation on the vertical axis, where the red dots represent upregulated proteins and the blue dots represent downregulated proteins (Figure 6B).

To confirm the stability and reproducibility of the proteomic results, a cluster analysis of the differentially expressed proteins was performed. As shown in Figure 6C,D, there were large differences between groups and small differences within groups, indicating high data accuracy suitable for subsequent analysis.

#### 3.5.3. Bioinformatics Analysis of Differential Proteins

**Figure 7:**
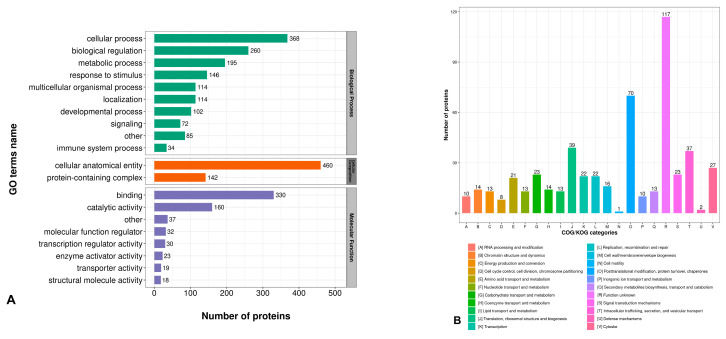

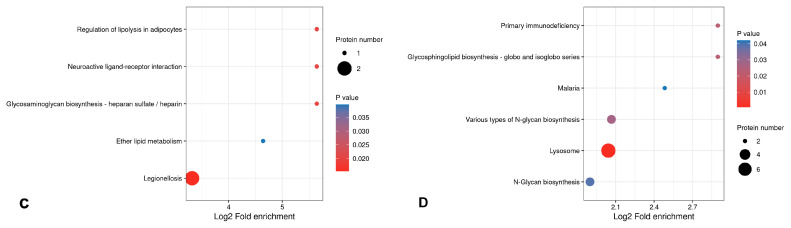
Results of bioinformatics analysis of differential proteins. (**A**) GO Level 2 annotation classification, (**B**) COG/KOG functional classification, and (**C**,**D**) KEGG enrichment.

In this experiment, a functional analysis of the differential proteins affected by SMSCs was performed using Lys. According to the GO annotation analysis of the differential proteins (Figure 7A), the categories of cellular process, biological regulation, metabolic process, responses to stimulus, and multicellular organismal process were significantly enriched in the biological process entry. The cellular anatomical entity and protein-containing complex were enriched in the cellular composition entry. The binding molecules and catalytic activity were enriched in the molecular function entry.

Figure 7B shows the functional classification, annotation, and prediction of differentially abundant proteins (DAPs) based on sequence similarity in the Clusters of Orthologous Groups (COGs) database. The COGs functional classification of DAPs is depicted in a bar graph with different colors representing different COGs classifications and the y-axis indicating the number of proteins in each category. The top five COGs functional classifications mainly included post-translational modifications/protein conversion/chaperone proteins, translation/ribosome structure and biogenesis, intracellular transport/secretion and vesicular transport, cytoskeletons, and signal transduction mechanisms.

In this study, Lys induced the proliferation and differentiation of SMSCs, which was accompanied by signal transduction and protein synthesis, resulting in changes in meat-quality traits.

The Kyoto Encyclopedia of Genes and Genomes (KEGGs) enrichment analysis (Figure 7C,D) showed that the upregulated differential proteins were mainly enriched in the pathways related to the regulation of adipocyte lipolysis, ether lipid metabolism, neuroactive ligand–receptor interactions, and glycosaminoglycan biosynthesis-heparan sulfate/heparin metabolism. The downregulated differential proteins were mainly enriched in the lysosome pathways. These results suggest that Lys may promote protein synthesis by stimulating SMSCs to proliferate and differentiate into adipocytes, thereby enhancing lipid metabolism.

#### 3.5.4. PPi Protein Interactions Analysis

**Figure 8:**
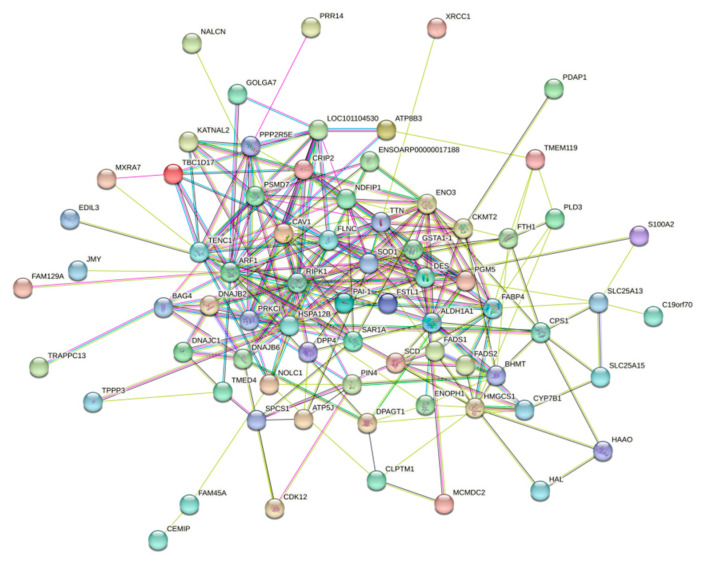
Protein interaction network.

Using the STRING website “https://cn.string-db.org (accessed on 20 December 2023)”, the interactions of 86 upregulated differential proteins were analyzed (Figure 8). These upregulated proteins were primarily concentrated in three major interaction networks: the lipid metabolism pathway, the sugar metabolism pathway, and the amino acid metabolism pathway.

In the interaction map, five proteins (Fatty acid-binding protein 4 (FABP4), 3-hydroxy-3-methylglutaryl-CoA synthase 1, HMGCS1 (HMGCS1), Stearoyl-CoA desaturase (SCD), Fatty acid desaturase 1 (FADS1), and Fatty acid desaturase 2 (FADS2)) were involved in lipid metabolism. Specifically, FADS2 and SCD are involved in lipid synthesis. Changes in the activity of SCD, an essential lipid-producing enzyme, can alter the rate of fatty acid biosynthesis, while FABP4 regulates lipolysis in adipocytes. Enolase 3 (ENO3) and Dipeptidyl peptidase-4 (DPP4) play roles in glucose metabolism, with ENO3 being significant for muscle development and regeneration. TTN and Creatine kinase S-type (CKMT2) are involved in amino acid metabolism, where CKMT2 primarily regulates skeletal muscle growth and development, and TTN is associated with muscle contraction.

#### 3.5.5. RT-qPCR Validation of Differentially Expressed Genes

To further validate the stability and reliability of the TMT-based quantitative proteomics data, five randomly selected, upregulated, differentially expressed genes (*SCD, FABP4*, *FADS1*, *FADS2*, and copper-zinc superoxide dismutase (*SOD1*)) were subjected to real-time fluorescent quantitative PCR assays.

**Figure 9:**
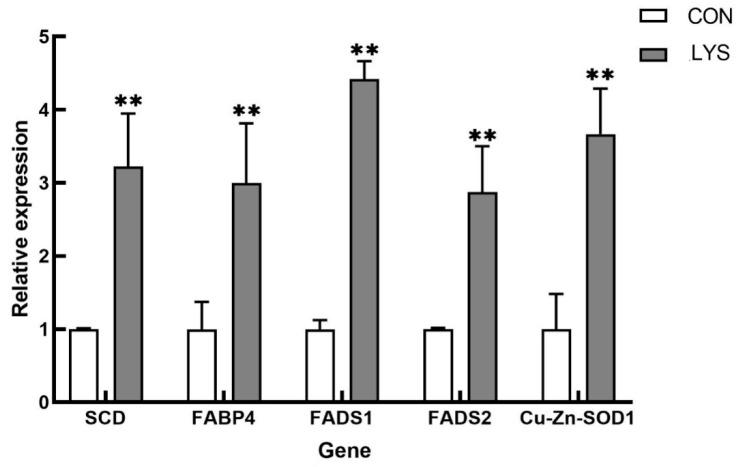
Real-time fluorescence quantitative PCR validation of differentially expressed proteins. “**” was considered *p* < 0.01.

As shown in Figure 9, the expression levels of these selected genes were consistent with the trends observed in the TMT quantitative proteomics results. This consistency indicates that the outcomes of the TMT-based quantitative proteomics sequencing are relatively stable and reliable.

## 4. Discussion

Lys may promote the proliferation and differentiation of SMSCs into myoblasts and lipoblasts by regulating the amino acid and lipid metabolism pathways. This regulation enhances protein synthesis, promotes the regeneration of muscle and fat [16], and improves skeletal muscle mass [17].

Previous studies have found that the members of the Glutathione Transferase (GSTA1-1) family present in SMSCs are novel modulators of Ryanodine Receptor (RyR) activity [18,19,20]. The RyR is located in the membrane of Ca^2+^ storage within the internal sarcoplasmic reticulum (SR) and is central to Ca^2+^ signaling and contraction in skeletal muscle. In Lys-induced SMSCs, RyR may be upregulated allosterically by mediating GSTA1-1, thereby protecting Ca^2+^ in skeletal muscle and the sarcoplasmic reticulum [21].

SOD1 is localized in the space between the cytoplasmic and mitochondrial membranes [22]. Previous studies have reported that SOD1 deficiency accelerates the accumulation of age-related muscle weight loss and oxidative damage in mice [23], suggesting that these effects are caused by SOD1 deficiency and the increased superoxidation of its secondary products. To maintain skeletal muscle homeostasis and repair damaged muscle, a group of specialized muscle stem cells (MuSCs) known as SMSCs activate, express myogenic transcription factors, migrate, multiply, and fuse with existing muscle fibers, or form new muscle fibers to complete regeneration. However, increased oxidative stress in muscle stem cells may impair the interaction between the existing muscle fibers and newly regenerated myoblasts and muscle stem cells, thereby impeding the muscle regeneration process [24].

SOD1 catalyzes the transformation of the superoxide free radical (O^2−^) into H_2_O_2_, playing a crucial role in the metabolism of oxygen free radicals. It catalyzes the initial reaction to remove oxygen free radicals, thereby preventing oxygen-induced cytotoxicity [25]. The upregulated differential protein SOD can enhance the expression of denodin and participate in muscle regeneration, likely due to SOD’s protective effect on the tissue membrane structure of skeletal muscle. This protective effect is attributed to SOD’s antioxidant properties. By reducing the content of peroxides in the cytosol and interstitial space of muscle tissue, SOD can promote regenerative processes, including protein synthesis [26].

FTH1 is a heavy subunit of ferritin, the main intracellular iron storage protein in both prokaryotes and eukaryotes. Its primary function is to store iron in a soluble and non-toxic state, playing a central role in maintaining cellular iron balance [27,28]. FTH1s are believed to facilitate the rapid detoxification of iron [29]. Changes in the composition of ferritin subunits can affect the rate of iron absorption and release in different tissues. It is crucial to store iron that does not require immediate metabolic use [30]. Iron is essential for effective muscle regeneration, highlighting the importance of iron homeostasis in this process [31].

CKMT2 has been shown to be an important protein involved in energy metabolism during muscle development in the tissues of pigs [32], cattle [33], chickens [34], and other species. CKMT2 acts on the arginine-proline metabolic pathway and participates in the embryonic development of skeletal muscle during the stage of muscle tube fusion to form muscle fibers. Upregulated CKMT2 increases creatine phosphate accumulation and ATP production because CKMT2 is involved in the mutual conversion of creatine and ATP to generate creatine phosphate (PCr) and ADP [35].

Among the upregulated differential proteins, SCD, FABP4, FADS1, and FADS2 are related to the transdifferentiation of SMSCs into adipocytes [36,37]. The result of Lys action is oxidation to produce an intermediate of the pyruvate, acetyl-CoA, or a tricarboxylic acid cycle, which can then be used in fatty acid synthesis to promote lipid production. One of the useful markers of lipogenic activity is SCD, an enzyme essential for the synthesis of monounsaturated fatty acids. SCD mainly produces palmitoleic acid and oleic acid, which are key substrates for complex lipids such as triglycerides, whose expression is upregulated up to 100 times during adipocyte differentiation [38]. Among the upregulated genes that regulate adipogenesis, we note the FABP family genes, which are key regulators of lipid synthesis and adipocyte differentiation. The FABP family was first discovered in the intestinal mucosa of rats in 1972 and includes FABP3, FABP4, FABP5, and FABP7 [39]. The protein coding of the *FABP* gene can bind to the hydrophobic ligands of long-chain fatty acids, promote the transmembrane transport of plasma fatty acids to the synthesis sites of triglycerides or phospholipids in cells, and promote fat accumulation [40,41,42]. In previous studies, FABP3, FABP4, and FABP5 were found to be continuously expressed at high levels during the M and L stages of transdifferentiation. Additionally, the expression of FABP4 was higher than that of the other FABP genes. This suggests that FABP genes, especially FABP4, can induce intracellular lipid droplet formation and accumulation in the late stages of the transdifferentiation of SMSCs [43].

In the KEGG pathway analysis, we found that some significantly downregulated proteins (LAMP1, LAMP2, WDR41, RRAS2, ATG12, GABARAP, ATG2B) and the upregulated protein ATG4B were enriched in the autophagolysosomal pathway. Autophagy, as the main mechanism of protein and cytoplasmic organelle degradation, is becoming a key regulator of muscle regeneration that affects stem cells, immune cells, and muscle fiber functions. A large number of extracellular stimuli (hormones, amino acids, or drugs) as well as intracellular stimuli (the invasion of microorganisms, the accumulation of misfolded proteins) can regulate the autophagy response. In mammalian cells, autophagy is a fundamental biological event that occurs under normal growth conditions [44]. One study showed that Lys supplementation inhibited autophagy activity and reduced muscle protein loss in aging mice [45]. Lys can reduce the degradation of certain muscle fibrils and maintain the stability of body proteins by inhibiting the autophagosome–lysosome system [46]. There are a range of mechanisms whereby extracellular and/or intracellular signals can be received and transmitted to regulatory factors to promote or inhibit autophagy when needed.

GABARAP is generally considered a member of the ATG8 family and has similar functions to ATG12, mainly involving the formation of autophagosomes and located on the septum of the autophagosome [47]. The cleavage of the C-terminal residues of ATG8 family proteins is mediated by ATG4 family proteases, which have four homologues (ATG4A-D) in mammals, among which ATG4B plays a major role in autophagy [48]. Recent evidence suggests that, in mammalian cells, the catalytic overexpression of ATG4B inhibits autophagy by sequestering non-lipid ATG8 homologues. The accumulation of overexpressed mutant ATG4Bs and the unturned autophagosome progenitors in these cells suggests that the lipidation of ATG8 homologous proteins is necessary for the formation of autophagosomes in mammalian cells [49]. In previous studies, Velikkakath et al. observed the accumulation of unsealed autophagosome-associated membranes in cells lacking ATG2B, suggesting impaired autophagosome closure [50]. Bozic et al. reported that the interaction between ATG2 and GABARAP is critical for IM closure. They found a highly conserved LC2 interaction region (LIR) in ATG2B, which mediates the interaction between ATG2 and GABARAP proteins [51]. Therefore, we can assume that under the stimulation of Lys as a nutrient, the upregulation of ATG4B is catalyzed, and the interaction between GABARAP, a homolog of ATG8, and ATG2B is inhibited, thus reducing the formation of autophagosomes and the closure of IM, thereby inhibiting protein degradation by the autophagy pathway. CMA is one of several lysosomal pathways for protein hydrolysis. In this pathway, a protein is pulled into the lysosomal cavity. During lysosomal fusion, phagocytic proteins are degraded after the fusion of the autophagosome and lysosome. Studies have shown that, in mammals, LAMP1 and LAMP2 play important roles in this process. In this study, Lys may inhibit the fusion of the autophagy and lysosome systems by downregulating LAMP1 and LAMP2, resulting in the inhibition of autophagy [52].

In the KEGG pathway analysis, we found that some downregulated proteins (GSDMD, SHARPIN, IL18, LOC101106452, IRAK4, ATG12, GABARAP) and the upregulated protein RIPK1 were enriched in the NOD-like receptor signaling pathways. As mentioned earlier, Lys has an inhibitory effect on autophagy. In the NOD-like receptor signaling pathway, Lys may have anti-inflammatory and anti-pyrogenic functions. Pyroptosis is the programmed death of inflammatory cells caused by an inflammasome. During pyroptosis, cytoplasmic pattern recognition receptors, apoptosis-associated spot-like proteins, and procysteine proteinase-1 form activated inflammasomes. Inflammasome-activated Caspase-1 leads to the production of the N-terminal cleavage product of gasdermin D (GSDMD), which is the primary executor of pyroptosis. As a result of pyroptosis, a large number of pro-inflammatory cytokines, including IL-1β and IL-18, are released [53]. In this study, the addition of Lys inhibited the production of GSDMD, thereby inhibiting the release of the pro-inflammatory factor IL-18, and thus inhibiting pyroptosis.

According to the information provided by the protein–protein interaction (PPI) network, the upregulated differential proteins are mainly concentrated in the lipid metabolism pathway, glucose metabolism pathway, and amino acid metabolism pathway. This is consistent with the analysis results of GO and KEGG. At the same time, we found some key genes in the PPI network, among which FLNC is at the core. Therefore, FLNC may play an important role in Lys-induced SMSCs, but studies on this have not been reported. Filamin C (FLNC) is a member of the actin-binding protein family [54], which plays a crucial role in the cytoskeleton and extracellular matrix (ECM) to maintain skeletal muscle integrity and function [55].

In summary, this study used Lys to induce the proliferation and differentiation of Sunit sheep SMSCs. The quantitative proteomic analysis showed that 86 proteins were upregulated, and 491 proteins were downregulated under Lys stimulation. The GO term enrichment of differential proteins showed a significant enrichment of the metabolic response and the stimulus response. The KEGG pathway analysis indicated that the autophagy–lysosome pathway was downregulated, inhibiting proteolysis after the Lys addition. The purpose of this study was to investigate the effect of Lys on SMSCs and its regulatory mechanism, providing data support for the subsequent amino acid optimization of mutton quality.

## 5. Conclusions

Lys can promote the proliferation of SMSCs. At the gene and protein levels, Lys promotes the expression of myogenic differentiation genes and proteins, indicating that Lys enhances the differentiation of SMSCs. The TMT quantitative proteomics analysis shows that Lys has antioxidant properties, maintains muscle stability, and promotes amino acid metabolism; simultaneously, Lys may also promote adipogenesis in SMSCs. The KEGG pathway analysis suggests that Lys may have anti-inflammatory, anti-pyroptotic, and proteolysis-inhibitory functions.

## Figures and Tables

**Table 1:** Real-time fluorescence quantitative PCR primer information.

Target Genes	Primers Sequences	Length of Product/Bp	GenBank Accession No.
*SCD*	F ACCATCACAGCACCTCCTTCC R CTCATTTCAGGGCGGATGTCTTC	98	NM_001009254.1
*FABP4*	F AAGAAGTGGGTGTGGGCTTTGC R TCCTGGCCCAATTTGAAGGACATC	145	NM_001114667.1
*FADS1*	F CCTGTGTTCCGTGCTGCTCAG R TGGTTCCACTTAGAGGTCCCGAAG	99	XM_004019593.6
*FADS2*	F GGAAGACTGCTGAGGACATGAACC R GATAGTGAACCAGGCGATGCTCTC	104	XM_015103138.4
*[Cu-Zn]-SOD1*	F GCCGTCTGCGTGCTGAAGG R CCAAACTGATGGACGTGGAACCC	137	NM_001145185.2
*β-actin*	F CCACAGCCGAGCGGGAAATTG R AGGAGGACGACGCAGCAGTAG	99	XM_004013078.5

## Data Availability

The data can be provided by the corresponding author on reasonable request.
